# Influence of *ABCB1* polymorphisms on aripiprazole and dehydroaripiprazole plasma concentrations

**DOI:** 10.1038/s41598-024-84192-8

**Published:** 2025-01-09

**Authors:** Francisco José Toja-Camba, María Vidal-Millares, María José Durán-Maseda, Gonzalo Hermelo-Vidal, Ángel Carracedo, Olalla Maroñas, Luis Ramudo-Cela, Irene Zarra-Ferro, Anxo Fernández-Ferreiro, Cristina Mondelo-García

**Affiliations:** 1 Pharmacy Department, University Clinical Hospital of Santiago de Compostela (SERGAS), 15706 Santiago de Compostela, Spain; 2 FarmaCHUSLab Group, Health Research Institute of Santiago de Compostela (IDIS), 15706 Santiago de Compostela, Spain; 3 Pharmacology, Pharmacy and Pharmaceutical Technology Department, Faculty of Pharmacy, University of Santiago de Compostela (USC), Santiago de Compostela, Spain; 4 Psychiatry Department, University Clinical Hospital of Santiago de Compostela, Santiago de Compostela, Spain; 5 Genetics Group, Health Research Institute of Santiago de Compostela (IDIS), Santiago de Compostela, Spain; 6 Galician Foundation of Genomic Medicine, Foundation of Health Research Institute of Santiago de Compostela (FIDIS), SERGAS, Santiago de Compostela, Spain; 7 Centre for Biomedical Network Research On Rare Diseases (CIBERER), Carlos III Health Institute, Madrid, Spain; 8 Pharmacogenomics and Drug Discovery (GenDeM), Foundation of Health Research Institute of Santiago de Compostela (FIDIS), Galicia, Santiago de Compostela, Spain; 9 Pharmacy Department, University Clinical Hospital A Coruña (CHUAC), A Coruña, Spain; 10 Hospital Pharmacy Research Group, Health Research Institute of A Coruña (INIBIC), A Coruña, Spain

**Keywords:** P-glycoprotein, Aripiprazole, Dehydroaripiprazole, Pharmacogenetics, Pharmacokinetics

## Abstract

Aripiprazole (ARI) is an atypical antipsychotic which is a substrate of P-glycoprotein (P-gp), a transmembrane glycoprotein that plays a crucial role in eliminating potentially harmful compounds from the organism. ARI once-monthly (AOM) is a long-acting injectable form which improves treatment compliance. Genetic polymorphisms in *ABCB1* may lead to changes in P-gp function, leading to individual differences in drug disposition. The present study aims to determine how the different variants of the three most prevalent SNPs of the *ABCB1* gene affect plasma concentrations of ARI, of its active metabolite dehydroaripiprazole (DHA) and ARI/DHA ratio in patients under AOM treatment. The metabolizing state of the two main aripiprazole metabolizing enzymes (CYP2D6 and CYP3A4) were considered to specifically study the effect of P-gp on plasma concentrations of the parent compound and its active metabolite. The study found a clear relationship between the genotypes found for the different *ABCB1* SNPs and the ARI/DHA ratio. Specifically, patients with GG genotype in G2677T have almost twice the ratio compared to TT genotype. Similarly, this increase is also found in C3435T with 1.4-fold and in C1236T with 1.6-fold for the same genotypes. Regarding haplotypes, significant differences were obtained between CC-GG-CC and TT-TT-TT patients, with an 87.9% higher ratio in patients with the CC-GG-CC haplotype. There was a clear trend towards lower ARI concentrations and higher DHA concentrations when the presence of mutated T alleles increases. The *ABCB1* gene could be a good partner along with CYP2D6 and CYP3A4 genotyping in conjunction with monitoring ARI plasma concentrations.

**Supplementary Information:**

The online version contains supplementary material available at 10.1038/s41598-024-84192-8.

## Introduction

Psychiatrists face significant difficulty in predicting the ideal dosage without objective metrics like antipsychotic plasma concentrations due to wide variations between individuals. This trial-and-error approach may delay reaching treatment goals and increase the risk of relapse or adverse reactions, which are closely linked with poorer prognosis and reduced treatment adherence (24,25).

Aripiprazole (ARI) is an atypical antipsychotic whose efficacy may be mediated through a combination of partial agonist activity at D2 and 5-HT_1A_ receptors and antagonist activity at 5-HT_2A_ receptors^1,2^. Aripiprazole once-monthly (AOM) is the long-acting injectable form, that improves the treatment compliance of this drug. ARI is metabolized through cytochrome CYP2D6 and CYP3A4 to its active metabolite dehydroaripiprazole (DHA)^3^. The polymorphic nature of *CYP2D6* and *CYP3A4* contribute to its plasma concentrations inter-individual variability^3^.

There is a consensus on the therapeutic range for both ARI (100–350 ng/mL) and active moiety (AM) (sum of ARI AND DHA) (150–500 ng/mL)^4^. Considering that there is available a target plasma concentration and the evidence that exists on the influence of the metabolizing state of CYP2D6 and CYP3A4 on these concentrations (dose recommendations included in Summary of product characteristics), the influence on aripiprazole plasma concentrations of another important player in the pharmacokinetics of numerous drugs in the body, P-Glycoprotein (P-gp), needs to be further explored.

P-gp is a transmembrane glycoprotein, first described in 1976, which plays a crucial role in the defense against potentially harmful compounds for the organism, including a variety of drugs^5^. Its location in the cell membrane, as an efflux transporter pump, allows it to actively transport drugs from the intracellular space to the extracellular environment, thereby affecting both plasma and tissue concentrations of pharmaceutical agents^6^.

The strategic placement of P-gp across various locations such as blood–brain-barrier (BBB), gut lumen, and within the renal and hepatic systems that underscores its integral role in drug metabolism and clearance, ensuring that xenobiotics and other substances are effectively removed from the body^7^.

P-gp is encoded by the human *ABCB1* gene, also known as Multridug Resistance protein 1 (*MDR*-*1*), which is located in chromosome 7^8^. In this sense, genetic polymorphisms in *ABCB1* may lead to changes in P-gp expression or function, leading to individual differences in drug disposition^9^. Specifically, among the reported single nucleotide polymorphisms (SNPs) in the *ABCB1* gene, there are three variants which have been the most thoroughly studied, C1236T (rs1128503) in exon 12, G2677T (rs2032582) in exon 21 and C3435T (rs1045642), in exon 26. These variants show strong linkage disequilibrium and were found to be functionally significant and ethnically distinct when associated with this region of the gene^10–14^. It is suggested that the higher the number of T alleles present, the lower the function or expression of P-gp^15^.

The influence of drug-drug interactions on plasma drug concentrations due to P-gp inhibitory drugs have been extensively studied^16^. Mostly, these studies evaluate the bioavailability of orally administered drug as a function of P-gp activity in the intestine^17^ or the outcomes in cancer treatment due to P-gp activity in tumor derived tissue^18,19^.

There are many drugs which are substrates of P-gp, so that variations in its function or expression can lead to alterations in their plasma concentrations and, consequently, affect their efficacy and safety^20^.

The first study to find differences in plasma drug concentrations depending on the *ABCB1* polymorphisms presented was developed by Hoffmeyer et al., reflecting the impact of polymorphisms in exon 26 (C3435T) on digoxin pharmacokinetics^21^. In addition, the inhibition of P-gp located in liver and kidney due to concomitant drugs has been evaluated by Taskar et al., who found increases in systemic concentrations of the P-gp substrates tested due to concomitant P-gp inhibitors^7^.

Regarding ARI and DHA, it has been proven that both are substrates of P-gp^22^. Specifically, in patients under oral aripiprazole treatment, differences in plasma concentrations of ARI due to *ABCB1* polymorphisms have been found^15^. To our knowledge, our study is the first to explore the influence of *ABCB1* polymorphisms on plasma concentrations in patients under AOM treatment.

The present study aims to determine how the different variants of the three most prevalent SNPs of the *ABCB1* gene affect plasma concentrations of ARI and DHA, and ARI/DHA ratio in patients under AOM treatment.

## Methods

An observational study of a cohort of patients under AOM treatment was conducted to evaluate the effect of the most prevalent *ABCB1* genetic polymorphisms on plasma concentrations of ARI and DHA. The study included outpatients who received at least four doses of AOM (Abilify Maintena; Otsuka, Tokyo, Japan) where adherence is fully ensured and documented in the electronic medical record. Exclusion criteria were age < 18 years, pregnancy, and cognitive impairment. Three blood samples were obtained from each patient, one for concentration analysis, one for genotyping and one for evaluation of biochemical data. Demographic characteristics and biochemical data including gender, age, height, weight, dosage, liver, and kidney function were recorded from the electronic medical record and were proactively contrasted with the patient. Also, a strict search for concomitant inhibitors and inducers for CYP2D6, CYP3A4 and P-gp was done taking into account the FDA list for this purpose^20^.

The authors declare that all procedures performed were in accordance with the ethical standards of the relevant national and institutional committees on human experimentation and with the 1975 Declaration of Helsinki (as revised 2008). All procedures involving patients were approved by the Drug Research Ethics Committee of Galicia (2020/486) and written informed consent was obtained from all subjects. It was signed by the patients if they had decision-making capacity; otherwise, it was signed by their legal guardians.

### Concentration analysis

Blood samples for pharmacokinetic analysis were collected in EDTA tubes immediately prior to AOM administration (Ctrough). All samples were centrifuged at 3.500 rpm for 10 min at 4 ◦C to obtain the plasma. A volume of 180uL of plasma sample was spiked with 20ul of aripiprazole-d8 (internal standard) and 500uL of acetonitrile and centrifuged. The supernatant was evaporated to dryness and the dry residue was reconstituted with 500uL of 10 mM Ammonium Formate/ Acetonitrile (85:15). Concentrations of ARI and DHA in plasma were measured with an ultra-high performance liquid chromatography-tandem mass spectroscopy (UHPLC-MS/MS) Xevo TQD triple quadrupole mass spectrometer (Waters, Massachusetts, USA), using a validated method previously published by the authors^23^.

### Genotype and phenotype

Genetic analysis was performed for SNPs for *ABCB1* gene. Analyses were performed using Taqman assays on the QuantStudio 12 K Flex (Applied Biosystems, Foster City, California). For *ABCB1*, the following variants were analyzed: C3435T (rs1045642), C2677 T/A (rs2032582) and C1236T (rs1128503). Also, due to the influence of CYP2D6 and CYP3A4 metabolizer status in ARI and DHA concentrations, the genotypes and phenotypes for these cytochromes were extracted from electronic clinical records. Phenotypes were stablished by assigning each allele an activity value corresponding to the activity value described by Caudle et al.^24^.

To avoid biases in the interpretation of the results, due to the demonstrated influence of the metabolizing phenotype of these cytochromes on plasma aripiprazole concentrations, only patients with normal phenotype metabolizer state for *CYP2D6* and absence of alleles *22 and *20 for CYP3A4 were ultimately selected. In addition, the presence of concomitant inhibitors or inducers for CYP2D6, CYP3A4 and P-gp were taken into account for patient screening.

The relationship between *ABCB1* and ARI and DHA concentrations have been evaluated for the three SNPs individually, in pairs and as haplotypes. In addition, patients with mutations in any of the SNPs were grouped as T-carriers and compared with those who were Non-T carriers for each SNP.

### Statistical analysis

Adjustment to a normal distribution was not assumed in concentration for ARI or DHA and ratios variables (Kolmogorov–Smirnov with Lilliefors correction normality test), so nonparametric tests were carried out. For comparison of two independent samples the Mann–Whitney tests were performed and for comparison of more than two independent samples the Kruskal–Wallis test were used to test the significance of differences between the different *ABCB1* groups. P values of less than or equal to 0.05 were considered to be statistically significant. Data were presented as median and interquartile ranges.

## Results

### Study population

A total of 72 patients under AOM treatment were recruited. Median age was 47 years (35–54); Weight 80.43 kg (67.3–91); BMI 29.1 kg/m^2^; Cr: 0.82 mg/dL (0.7–0.97); AST: 23 IU/L (19–30.5); ALT: 30 IU/L (18–42); GGT: 27 IU/L (17–42); Median dose administrated of AOM was 400 mg (300–400); Injection site (28 Deltoid; 44 Gluteus); smoking status (49 smokers; 23 non-smokers). Since only one of the patients included in the study was on treatment with 300 mg doses, correction for dose concentrations was not performed for the analysis. With respect to active moiety 58/72 patients were within the therapeutic range. The median concentration of DHA with respect to ARI was 44.8% (30%-55%) and of DHA with respect to AM was 30.9%(23.1%-35.5%). The genotypes and phenotypes of *CYP2D6* and *CYP3A4* are depicted in Table 1. Finally, after considering only patients with normal phenotype metabolizer state for *CYP2D6* and absence of alleles *22 and *20 for CYP3A4 together with the absence of concomitant inhibitors or inducers, a total of 35 patients were selected. The distribution of *ABCB1* genotypes for these 35 patients is shown in Table 2.

**Table 1:** CYP3A4 presence of *22 and *20 alleles detected, CY2D6 Phenotypes and *ABCB1* single nucleotide polymorphisms (SNPs) variants and haplotypes.

	SNP	n (%)	Haplotype	n (%)
C3435T	CC	23 (31.9)	CC-GG-CC	10 (13.9)
	CT	34 (47.2)	CC-GA-CC	2 (2.8)
	TT	15 (20.8)	CC-GT-CC	1 (1.4)
G2677T	GG	22 (30.6)	CC-GG-CT	4 (5.6)
	GT	36 (50)	CC-GG-TT	3 (4.2)
	TT	11 (15.3)	CC-GA-TT	1 (1.4)
	GA	3 (4.2)	CC-GT-CT	2 (2.8)
C1236T	CC	19 (26.4)	CT-GG-CC	2 (2.8)
	CT	33 (45.8)	CT-GG-TT	3 (4.2)
	TT	20 (27.8)	CT-GT-CC	1 (1.4)
	PM	2 (2.7)	CT-GT-CT	20 (27.8)
*CYP2D6* Phenotype	IM	27 (37.5)	CT-GT-TT	5 (6.9)
	NM	40 (55.5)	CT-TT-CC	1 (1.4)
	UM	3 (4.1)	CT-TT-TT	2 (2.8)
*CYP3A4*	*1/*22	3 (4.1)	TT-GT-CT	7 (9.7)
Genotype	*22/*22	2(2.7)	TT-TT-CC	2 (2.3)
			TT-TT-TT	6 (8.3)

**Table 2:** *ABCB1* single nucleotide polymorphisms (SNPs) variants and Haplotypes after selection of normal metabolizing states for CYP2D6 and absence of alleles *22 and *20 for CYP3A4.

	SNP	n (%)	Haplotype	n (%)
C3435T	CC	15 (42.9)	CC-GG-CC	8 (22.9)
	CT	14 (40)	CC-GA-CC	2 (5.7)
	TT	6 (17.1)	CC-GG-CT	1 (2.9)
G2677T	GG	13 (37.1)	CC-GG-TT	2 (5.7)
	GT/GA	18 (51.4)	CC-GA-TT	1 (2.9)
	TT	4 (11.4)	CC-GT-CT	1 (2.9)
C1236T	CC	12 (34.3)	CT-GG-CC	1 (2.9)
	CT	16 (45.7)	CT-GG-TT	1 (2.9)
	TT	7 (20)	CT-GT-CT	12 (34.3)
			TT-GT-CT	2 (5.7)
			TT-TT-CC	1 (2.9)
			TT-TT-TT	3 (8.6)

### Effect of variations in individual *ABCB1* SNPs

A comparative analysis of the concentrations of ARI, DHA as well as the ratio of their plasma concentrations (ARI/DHA ratio) between the different variants found was carried out (Table S1).

For ARI concentrations, no significant differences were found between the different variations for the three SNPs analyzed. However, there was a clear trend across the three SNPs to lower ARI concentrations as the presence of mutated T alleles increases.

On the other hand, in the case of DHA concentrations, there was also a trend towards higher DHA concentrations the higher the number of T alleles. Specifically, in the case of C1236T significant differences (p < 0.05) were found with 59.9% higher DHA concentrations for CT (68.99 ng/mL) compared to CC (43.13 ng/mL).

**Fig. 1:**
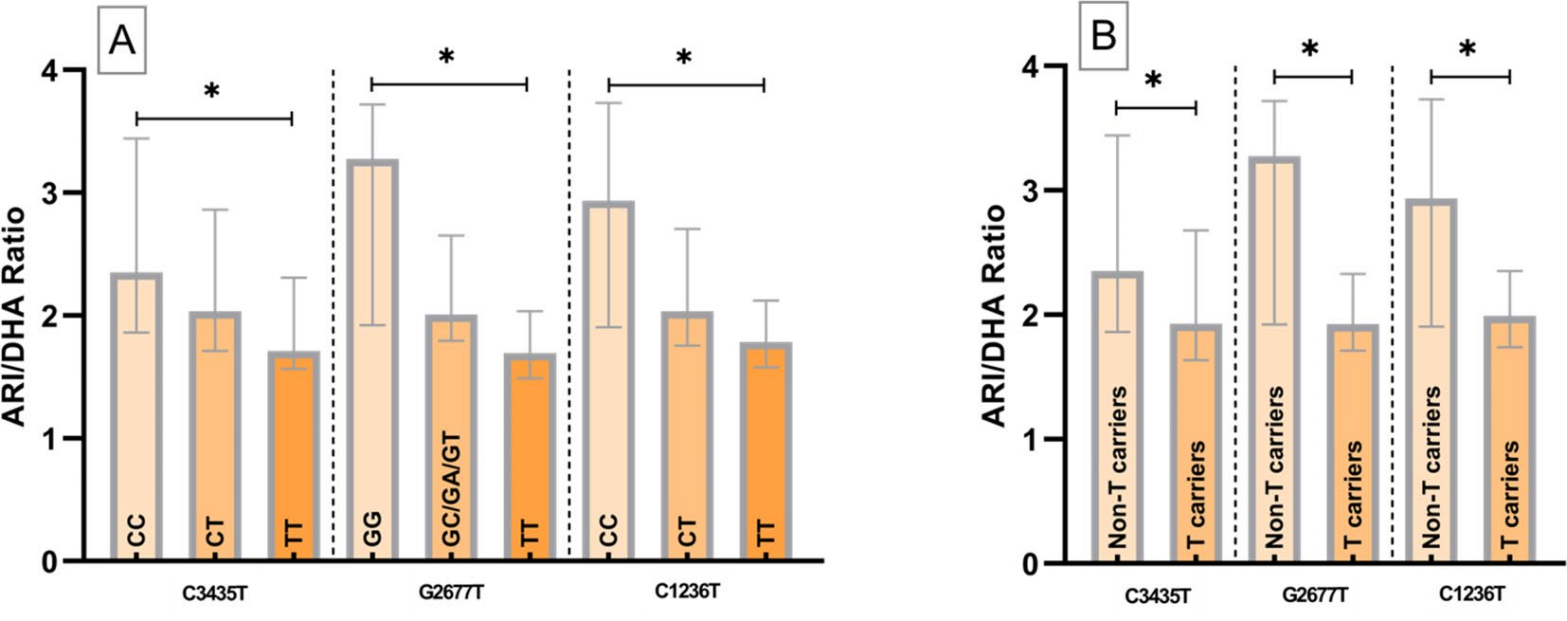
Aripiprazole/dehydroaripiprazole (ARI/DHA) ratio among the different *ABCB1* single nucleotide polymorphisms (SNPs) variants and comparison between Non-T carriers vs T carriers for each SNP; *(p < 0.05).

Regarding ARI/DHA ratio, significant differences between the different variants were observed in the three SNPs analyzed. Specifically, for C3434T it was obtained an ARI/DHA ratio 37.4% higher for CC (2.35) compared to the TT variant (1.71), for G2677T 93% higher for GG (3.27) vs TT (1.69) and for C1236T 64.2% higher for CC (2.93) vs TT (1.78) (Fig. 1A). Comparing Non-T carriers vs. T carriers variants as a whole, yielded 22.3%, 70.07% and 47.43% more respectively, maintaining statistical significance (Fig. 1B).

### Effect of *ABCB1* haplotypes

**Fig. 2:**
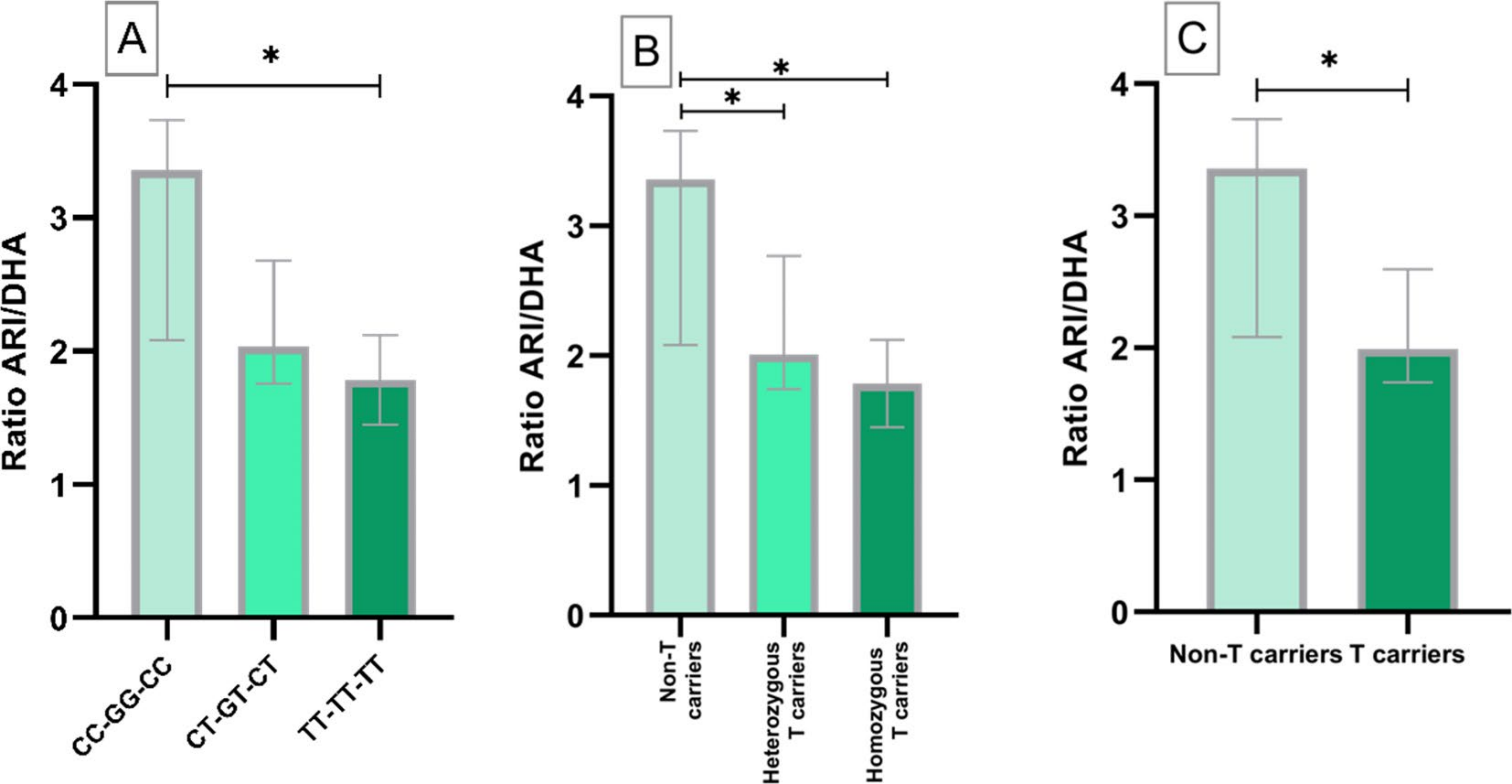
Aripiprazole/dehydroaripiprazole (ARI/DHA) ratio among the three different *ABCB1* haplotypes analyzed and comparison between T carriers and Non-T carriers; *(p < 0.05).

The different *ABCB1* haplotypes were analyzed together (Table S2). The 3 groups with the highest number of patients were used for the analysis. Regarding ARI and DHA concentrations, no significant differences were found between the three groups, but a slight trend towards higher ARI and lower DHA concentrations was observed with increasing presence of T alleles. Significant differences were obtained between CC-GG-CC and TT-TT-TT patients, with an 87.9% higher ratio in patients with the CC-GG-CC haplotype (Fig. 2A). Non-T carriers, heterozygous T carriers and homozygous T carriers patients were also compared. In this case, 67.09% higher ratios were obtained in Non-T carriers compared to heterozygous T carriers (p < 0.05) (Fig. 2B). This difference was maintained when comparing Non-T carriers to T-carriers (homozygous and heterozygous) (Fig. 2C).

**Fig. 3:**
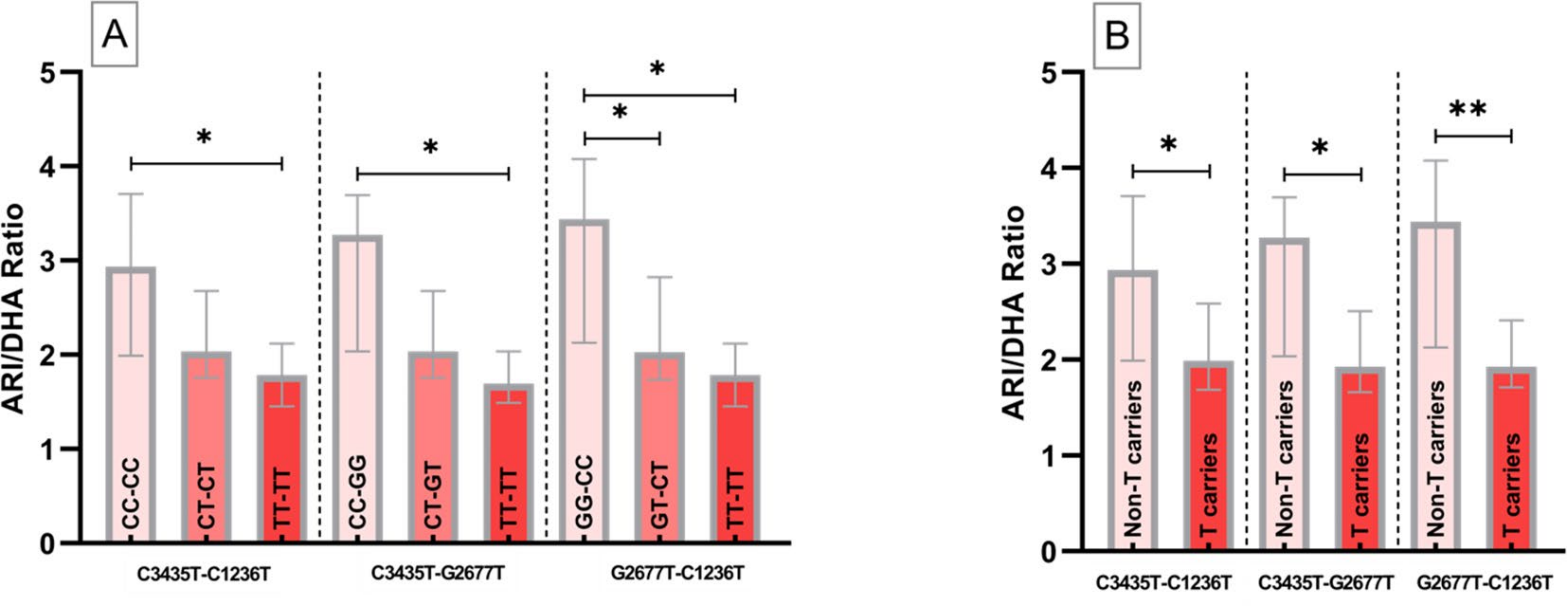
ARI/DHA ratio among the different paired *ABCB1* SNPs variants and comparison between T carriers and Non-T carriers. *(P < 0.05); **(P < 0.01).

To reinforce the results obtained and to confirm whether the observed differences were maintained when grouping the patients by pairs of variants of the three SNPs, the same comparative analyses were carried out for the pairs C3435T-C1236T, C3435T-G2677T and G2677-C1236T. Identical results were obtained for the rest of the comparisons performed, with even greater statistical significances (p < 0.01) (Fig. 3). The results of this analysis are shown in Table S3.

## Discussion

This study explores how three of the most studied SNPs of the *ABCB1* gene to date affect plasma aripiprazole concentrations in patients under AOM treatment for the first time. In addition, this study has considered the metabolizing state of the two main ARI metabolizing enzymes (CYP2D6 and CYP3A4), in order to eliminate biases due to metabolizer status of these cytochromes and to specifically study the effect of P-gp on plasma concentrations of the parent compound and its active metabolite. Regarding the percentage of DHA with respect to ARI, we have obtained a higher percentage than those reported in the summary of products characteristics (SmPC). This could be due to different causes, such as uncontrolled external factors or that the SmPC does not take into account the variability of metabolizing genotypes. The allele frequencies for the *ABCB1* gene obtained in our study population were similar to those of the reference population^25,26^.

In psychiatry, advances on the implementation of pharmacogenetics in clinical practice depend on the integration of genetic biomarkers along with other relevant parameters and clinical information influencing variability in drug response. To determine the clinical consequences of having certain alleles, a joint interpretation with other parameters is required. This allows for interpretation and translation into clinical decision-making to optimize drug treatment.

Our results show a clear relationship between the genotypes found for the different *ABCB1* SNPs and the ARI/DHA ratio. Individually, patients with GG genotype in G2677T have almost twice the ratio compared to TT genotype. Similarly, this increase is also found in C3435T with 1.4-fold and in C1236T with 1.6-fold for the same genotypes. These results are reinforced by comparing the three haplotypes analyzed and obtaining significant differences between Non-T carriers and T carriers.

Other previous studies analyzed some of these SNPs individually, but not haplotypes, nor did they consider CYP2D6 or CYP3A4 genotypes to select patients. Specifically, *Suzuki *et al*.* observed trends to lower ARI concentrations for TT-TT genotype of C2677T and C3435TT but no significant differences were found^27^. In contrast, *Rafaniello *et al*.* found significant differences in patients with TT genotype for these SNPs, in whom they observed lower ARI concentrations^28^. These results are in agreement with those obtained in this work, in which T homozygous carriers patients for the three SNPs individually show a tendency towards lower ARI concentrations. On the other hand, another work developed by *Belmonte *et al*.* found higher ARI concentrations in those patients with the presence of T alleles^15^. These studies were performed with oral ARI, so part of the observed effect was artifacted due to the influence of intestinal P-gp. In contrast, this work was performed with AOM, so the contribution of intestinal P-gp should be minimal, focusing on the effects caused by the different variants of the polymorphisms in the other tissues where P-gp is present.

Regarding DHA concentrations, higher values were found as the presence of T alleles increases both for the SNPs individually and for the haplotypes studied. In particular, a greater influence is observed in DHA concentrations than in the parent compound, being its greater P-gp affinity comparing to ARI one of the possible causes (21).

The effect of the different P-pg polymorphisms produce significant differences in ARI/DHA ratio. However, regarding plasma concentrations of ARI and DHA, despite there is a clear trend towards lower ARI concentrations and higher DHA concentrations when the presence of mutated T alleles increases, no significant differences were found. This may be due to the different hepatic P-gp activity between patients, as well as to the aforementioned different P-gp affinity for both substrates. Although the pharmacological activity of DHA seems to be similar to that of ARI, it would be necessary to study whether these differences in the ratio, in favour of DHA, produce a decrease in the time in which the therapeutic effect is maintained, due to the shorter half-life of this metabolite compared with the parent compound^29^.

Regardless of whether or not it affects the active moiety of plasma concentrations, both ARI and DHA are obvious substrates of P-gp, and therefore at the BBB these polymorphisms may predispose to lower drug effectiveness or greater adverse effects, this hypothesis is reinforced by differences at this level, found by Kirschbaum et al. and Wang et al., in P-gp knockout animal models^22,30^. Regarding limitations, this study, which is limited by a reduced sample size, focuses on the three most studied SNPs of the *ABCB1* gene in the ARI/DHA ratio, neglecting additional external factors that may influence plasma concentrations, as well as the potential influence of rarer *ABCB1* polymorphisms that have not been explored. These rarer variants might hold significant weight in individual responses to medications, and their absence from the analysis could lead to an incomplete picture of the impact of *ABCB1* on aripiprazole pharmacogenetics^31^.

In conclusion, the *ABCB1* gene could be a good partner along with *CYP2D6* and *CYP3A4* genotyping in conjunction with monitoring plasma concentrations of aripiprazole. Further studies should evaluate clinical efficacy and adverse effects, together with analysis of the different *ABCB1* SNPs, as well as CYP2D6 and CYP3A4 genotype, to clarify the clinical impact of these three *ABCB1* polymorphisms.

## Electronic supplementary material

Below is the link to the electronic supplementary material.

Supplementary Material 1

## Data Availability

The datasets used and/or analysed during the current study available from the corresponding author on reasonable request.
