# Supplementary material for: Influence of ABCB1 polymorphisms on aripiprazole and dehydroaripiprazole plasma concentrations
Source: Sci Rep. 2025 Jan 9;15:1521. doi: 10.1038/s41598-024-84192-8 (PMC11717999; doi:10.1038/s41598-024-84192-8)
Supplement: Supplementary file 1 — Supplementary Material 1 [file 41598_2024_84192_MOESM1_ESM.doc]

| **SNP** | **Genotype (n)** | **ARI (ng/mL) (IQ)** | **DHA (ng/mL) (IQ)** | **ARI/DHA RATIO (IQ)** |
| --- | --- | --- | --- | --- |
| **C3435T** | **CC (15)** | 152.9 (98.4-187.4) | 56 (40.8-68.4) | 2.3***** (1.9-3.4) |
|  | **CT (14)** | 138.9 (105.5-178.9) | 62.5 (43.3-79.5) | 2 (1.7-2.9) |
|  | **TT (6)** | 117.5 (67.5-271) | 66.3 (41.5-114) | 1.7***** (1.6-2.3) |
|  | **T Carrier**  **(CT or TT) (20)** | 133.5 (101.2-181.5) | 65.5 (42.4-80.5) | 1.9***** (1.6-2.7) |
| **G2677T** | **GG (13)** | 152.9 (129.3-188.8) | 56 (40.4-66.7) | 3.3***** (1.9-3.7) |
|  | **GA/GT (18)** | 127 (97.7-183.9) | 62.5 (43.7-85.4) | 2 (1.8-2.6) |
|  | **TT (4)** | 117.5 (74.2-312.8) | 66.3 (46.6-173.5) | 1.7***** (1.5-2) |
|  | **T Carrier (**  **GT or TT) (22)** | 127 (97.7-183.9) | 65.5 (43.7-85.4) | 1.9***** (1.7-2.3) |
| **C1236T** | **CC (12)** | 151.1 (95.8-165.5) | 43.1**^+^** (34.6-64.2) | 2.9***** (1.9-3.7) |
|  | **CT (16)** | 138.9 (104.9-197.3) | 69**^+^** (54.3-85.2) | 2 (1.7-2.7) |
|  | **TT (7)** | 134.9 (98.38-187.4) | 63.6 (53.5-94) | 1.8***** (1.6-2.1) |
|  | **T Carrier**  **(CT or TT) (23)** | 134.9 (100-187.4) | 67.3***** (53.7-86.1) | 2***** (1.7-2.3) |

**Table S1.** Plasma concentrations of Aripiprazole **(**ARI) and dehydroaripiprazole (DHA) and ARI/DHA ratios among the different *ABCB1* single nucleotide polymorphisms (SNPs) variants and haplotypes. Results are presented as median and Interquartile Range (IQ); *****Significant difference between Non-T carriers and homozygous T carriers (p<0.05); **^+^**Significant difference between Non- T carriers and heterozygous T carriers (p<0.05).

| **Haplotype** | **ARI (ng/mL) (IQ)** | **DHA (ng/mL) (IQ)** | **ARI/DHA RATIO (IQ)** |
| --- | --- | --- | --- |
| **CC-GG-CC (8)** | 156.3 (137-201.7) | 59.5 (41.9-67.5) | 3.4***** (2-3.7) |
| **CT-GT-CT (12)** | 138.9 (108.5-181.5) | 69 (56.4-89.7) | 2 (1.7-2.7) |
| **TT-TT-TT (3)** | 134.9 (100-372.1) | 69 (63.6-208.3) | 1.8***** (1.4-2.1) |
| **Non-T carriers**  **(CC-GG-CC) (8)** | 156.3 (137-201.7) | 59.5 (41.9-67.5) | 3.4***^+^** (2.1-3.7) |
| **Heterozygous T carriers (24)** | 127 (96.3-181.5) | 56.8 (41.1-80.5) | 2**^+^** (1.74-2.76) |
| **TTT haplotype homozygous carriers (3)** | 134.9 (100-372.1) | 69 (63.6-208.3) | 1.8***** (1.4-2.1) |
| **Non- T carriers (8)** | 156.3 (137-201.7) | 59.5 (41.9-67.5) | 3.4* (2.1-3.7) |
| **T carriers (27)** | 132.1 (98.4-182.8) | 57.6 (41.7-82.5) | 2* (1.7-2.6) |

**Table S2.** Plasma concentrations of Aripiprazole (ARI) and dehydroaripiprazole (DHA) and ARI/DHA ratios among the different *ABCB1* haplotypes. Results are presented as median and Interquartile Range (IQ); *Significant difference between Non-T carriers and homozygous T carriers (p<0.05); +Significant difference between Non- T carriers and heterozygous T carriers (p<0.05). Heterozygous T carriers: CC-GA-CC; CC-GG-CT; CC-GG-TT; CC-GA-TT; CC-GT-CT; CT-GG-CC; CT-GG-TT; CT-GT-CT; TT-GT-CT; TT-TT-CC

|  | **SNP** | **ARI (ng/mL) (IQ)** | **DHA (ng/mL) (IQ)** | **ARI/DHA RATIO (IQ)** |
| --- | --- | --- | --- | --- |
| **C3435T -C1236T** | **CC-CC (10)** | 152.3 (116.3-178.8) | 51.2 (35.3-65.8) | 2.9***** (2-3.7) |
|  | **CT-CT (12)** | 138.9 (108.5-181.5) | 69 (56.4-89.7) | 2 (1.7-2.7) |
|  | **TT-TT (3)** | 134.9 (100.372.1) | 69 (63.6-208.3) | 1.8***** (1.4-2.1) |
|  | **T Carriers (25)** | 134.9 (99.19-185.1) | 63.6 (48.9-84.3) | 2* (1.7-2.6) |
| **C3435T-G2677T** | **CC-GG (11)** | 159.7 (132.1-202.2) | 57 (45.3-68.4) | 3.3* (2-3.7) |
|  | **CT-GT (12)** | 138.9 (108.5-181.5) | 69 (56.4-89.7) | 2 (1.7-2.7) |
|  | **TT-TT (4)** | 117.5 (74.17-312.8) | 66.3 (46.6-173.5) | 1.7* (1.5-2) |
|  | **T carriers (24)** | 127 (96.38-181.5) | 60.6 (41.1-80.5) | 1.9* (1.7-2.5) |
| **G2677-C1236T** | **GG-CC (9)** | 152.9 (141.3-190.3) | 57 (38.5-66.7) | 3.4*^+^ (2.1-4.1) |
|  | **GT-CT (15)** | 132.1 (104.6-182.8) | 67.3 (53.7-82.5) | 2^+^ (1.7-2.8) |
|  | **TT-TT (3)** | 134.9 (100-372.1) | 69 (63.6-208.3) | 1.8* (1.4-2.1) |
|  | **T carriers (26)** | 127 (97.7-183.9) | 60.63 (43.7-83) | 1.9** (1.7-2.4) |

**Table S3.** Plasma concentrations of aripiprazole (ARI) and dehydroaripiprazole (DHA) and Ratios ARI/DHA among the different paired *ABCB1* single nucleotide polymorphisms (SNPs) variants. Results are presented as median and Interquartile Range (IQ); *****Significant difference between Non-T carriers and homozygous T carriers (p<0.05); ******Significant difference between Non-T carriers and homozygous T carriers (p<0.01); **^+^**Significant difference between Non- T carriers and heterozygous T carriers.
